# Modelling kidney outcomes based on MELD eras - impact of MELD score in renal endpoints after liver transplantation

**DOI:** 10.1186/s12882-022-02912-6

**Published:** 2022-08-23

**Authors:** Paulo Ricardo Gessolo Lins, Roberto Camargo Narciso, Leonardo Rolim Ferraz, Virgilio Gonçalves Pereira, Ben-Hur Ferraz-Neto, Marcio Dias De Almeida, Bento Fortunato Cardoso Dos Santos, Oscar Fernando Pavão Dos Santos, Júlio Cesar Martins Monte, Marcelino Souza Durão Júnior, Marcelo Costa Batista

**Affiliations:** 1 Hospital Israelita Albert Einstein, São Paulo, Brazil; 2 Division of Nephrology, Federal University of São Paulo, São Paulo, Brazil; 3 Division of Nephrology, New England Medical Center, Tufts University, Medford, MA 02155 USA

**Keywords:** Acute Kidney Injury, Liver Transplantation, Liver Cirrhosis, MELD

## Abstract

**Background:**

Acute kidney injury is a common complication in solid organ transplants, notably liver transplantation. The MELD is a score validated to predict mortality of cirrhotic patients, which is also used for organ allocation, however the influence of this allocation criteria on AKI incidence and mortality after liver transplantation is still uncertain.

**Methods:**

This is a retrospective single center study of a cohort of patients submitted to liver transplant in a tertiary Brazilian hospital: Jan/2002 to Dec/2013, divided in two groups, before and after MELD implementation (pre-MELD and post MELD). We evaluate the differences in AKI based on KDIGO stages and mortality rates between the two groups.

**Results:**

Eight hundred seventy-four patients were included, 408 in pre-MELD and 466 in the post MELD era. The proportion of patients that developed AKI was lower in the post MELD era (*p* 0.04), although renal replacement therapy requirement was more frequent in this group (*p* < 0.01). Overall mortality rate at 28, 90 and 365 days was respectively 7%, 11% and 15%. The 1-year mortality rate was lower in the post MELD era (20% vs. 11%, *p* < 0.01). AKI incidence was 50% lower in the post MELD era even when adjusted for clinically relevant covariates (*p* < 0.01).

**Conclusion:**

Liver transplants performed in the post MELD era had a lower incidence of AKI, although there were more cases requiring dialysis. 1-year mortality was lower in the post MELD era, suggesting that patient care was improved during this period.

## Background

Liver transplantation (LT) is preferred therapy for individuals with advanced chronic liver disease and those with acute liver failure [1]. Disparities among grafts distribution and patient selection force transplant polices to draw a specific liver allocation criterion that include MELD score [2–6]. From the past 10 years, this score has been used by the UNOS, Euro transplant for prioritizing allocation of liver transplants and Brazilian liver allocation policy instead of the previous CPT score [2, 7, 8].

MELD score-based liver allocation policy continues under discussion, especially because some authors criticize that patient with remarkably high scores are too sicky to undergoing liver transplantation [8]. Other studies call attention to the increasing prevalence of hepatocellular carcinoma that could lead patients without this condition to receive a liver transplant late – because hepatocellular carcinoma diagnosis supplements MELD scores irrespective to laboratorial data [9]. Instead, this allocation policy can reduce the time on the waiting list for LT and mortality from all causes of the patient with end-stage liver disease [10, 11].

Patients undergoing LT frequently experience some degree of AKI through the perioperative period of LT [12]. This AKI could be explained by liver transplantation by itself plus additional insults to the kidney, such as hemorrhage [13], administration of nephrotoxic drugs [13, 14], post reperfusion syndrome [15], hypotension [16] and often substantial blood transfusion [17]. Although kidney injury is usually reversible, it implies some complications, such as increased length of hospitalization, longer mechanical ventilation time, sepsis, and progression to chronic renal failure, directly contributing to lower liver graft and patient survival [17, 18].

Serum creatinine is a cornerstone of AKI diagnosis in general population [12]. Despite interferences in end-stage liver disease and post LT, this test is still consistent for AKI diagnosis in these setting [19]. Also, MELD score employs serum creatinine as a variable, and it is known that end stage liver disease patient with AKI is more likely to receive a higher priority in transplantation list based on MELD score [20].

Few studies report time disparities in kidney disfunction after LT based on MELD score-based liver allocation policy implementation. Lee et al. failed to demonstrate differences between three ages (1996–2000 / 2001–2005 / 2006–2008) in identifying chronic kidney disease in a cohort of 431 patients after 6 months of LT [21]. Leithead et al. shows increase in incidence of AKI over 3 study periods (2000–2003, 2004–2007 and 2008–2011) even after adjusted for confounding variables [22]. However, this United Kingdom cohort adopts LT allocation policy with a different model – the UKELD score-based model for LT, a slightly different score compared with MELD score [5].

Considering the high incidence of AKI in LT recipients, we proposed this observational study to evaluate the impact of MELD score-based liver allocation policy implementation on AKI diagnosis, classification, RRT requirement, and mortality.

## Methods

This study was approved by local ethics committee of the Hospital Israelita Albert Einstein and informed consent were waived by the same committee—(number 00737118.6.0000.0071), São Paulo, Brazil. All methods were carried out in accordance with relevant guidelines and regulations’ or the ‘Declaration of Helsinki.

### Data collection

Data were collected from a series of liver transplants performed at the Hospital Israelita Albert Einstein, São Paulo, Brazil from January 2002 to December 2014 from living and deceased donors. Patients extracted until December 2006 were labelled as pre-MELD ERA, while patients after January 2007 as post MELD ERA. All transplantation recipients were admitted to the ICU in the immediate postoperative period. Before January 2007, the allocation of organs for liver transplantation was done by waiting time on the list, and subsequently started to be based on the MELD score [23]. This study adheres to the Strengthening the Reporting of Observational Studies in Epidemiology guidelines for the reporting of cohort studies – STROBE [24].

Medical records for patients undergoing LT from 2002 to 2014 were retrospectively reviewed to reclaim hospitalization clinical data, including baseline demographic characteristics and comorbidities, preoperative clinical and laboratory records, main indication of LT, liver disease stage according to the CPT and MELD score, and intraoperative variables including liver graft donor type, surgical time, total ischemia period, vasopressor, and transfusion requirement. Admission eGFR was calculated by MDRD equation as formerly recommended for cirrhotic patients [12].

A single surgical team, all particularly trained in LT, performed all procedures. The immunosuppressive routine lay on a calcineurin inhibitor (cyclosporine predominantly until 2005 and mainly tacrolimus later), an antiproliferative drug (mycophenolate) and a corticosteroid. Whole blood levels of calcineurin inhibitor were measured by fluorescence polarization immunoassay.

Postoperative variables included the development of sepsis, need for vasopressor drugs, use of well-recognized nephrotoxic agents, including radiocontrast agents, nonsteroidal anti-inflammatory drugs, and antimicrobials (vancomycin, aminoglycosides, polymyxin B and amphotericin B). APACHE II score or SAPS3 were determined at the time of admission to the ICU [25, 26]. MELD score was calculated according to the equation described by Kamath and coworkers [3, 4]. Sepsis was defined in conjunction with the international consensus definition [27]. Severe liver graft dysfunction included primary nonfunction and graft dysfunctions according to a former described criterion for liver failure [28, 29].

AKI was defined according to the AKI KDIGO recommendations [30]. Exact dates of AKI event and stage reached in seven days were obtained by computerized scanning of the results of daily laboratory tests for serum creatinine and 24-h urinary output from the electronic record system for each LT as the index event. RRT was initiated at the discretion of the nephrology team, based on common clinical indications such as hypervolemia, hyperkalemia, refractory acidosis, uremic sign, or symptoms, and/or anuria. All attending nephrologists were part of the same group. Patients who developed AKI before LT or required RRT prior to LT were excluded from this analysis, as well as those receiving simultaneous kidney and liver transplant or who underwent liver re-transplantation. The main predictor variable of interest for the primary outcome was the period which LT were performed (Before or after MELD score-based liver allocation policy implementation, Pre-MELD and Post MELD, respectively).

The primary outcome in this analysis was AKI development after LT. Secondary outcomes were death for any cause, RRT requirement, AKI stages and RRT duration.

### Statistical analysis

Numerical variables were labelled by median and interquartile range, and categorical variables by absolute and relative frequencies. For appraisal of baseline characteristics regarding the main predictor variable and primary outcome, univariable analysis was performed with the chi-squared test and Mann–Whitney U test for categorical and continuous variables, respectively.

Our data has two mortality risk scores collected at the time of ICU admission – APACHE2 for admissions until December 2011 and SAPS3 for admissions after that. In order to homogenize our data, a multiple imputation approach was used to create homogeneity among APACHE2 and SAPS3 scores using two groups in common demographic and before liver transplantation clinical data [31].

Also, a Receiver-Operating Curve (ROC) was performed to examine the discriminating power of APACHE2 and SAPS3 for mortality prediction. Additionally, ROC was performed with MELD for mortality, AKI and RRT requirement risk prediction.

Univariable analysis was performed to identify additional variables associated with primary outcome as potential confounders, with each variable in the database entered a logistic regression as a single covariate with AKI diagnosis as dependent variable. Those variables with a *p *value < 0.1 in univariable analysis or with undoubtedly clinical relevance were subsequently entered into a multivariable logistic model. A Cox proportional hazard model were performed within the main predictor (MELD era), clinical variables and AKI classification as covariates and one-year survival as independent variable. All tests were performed using SPSS version 26.0 (SPSS, Chicago, IL).

## Results

**Fig. 1:**
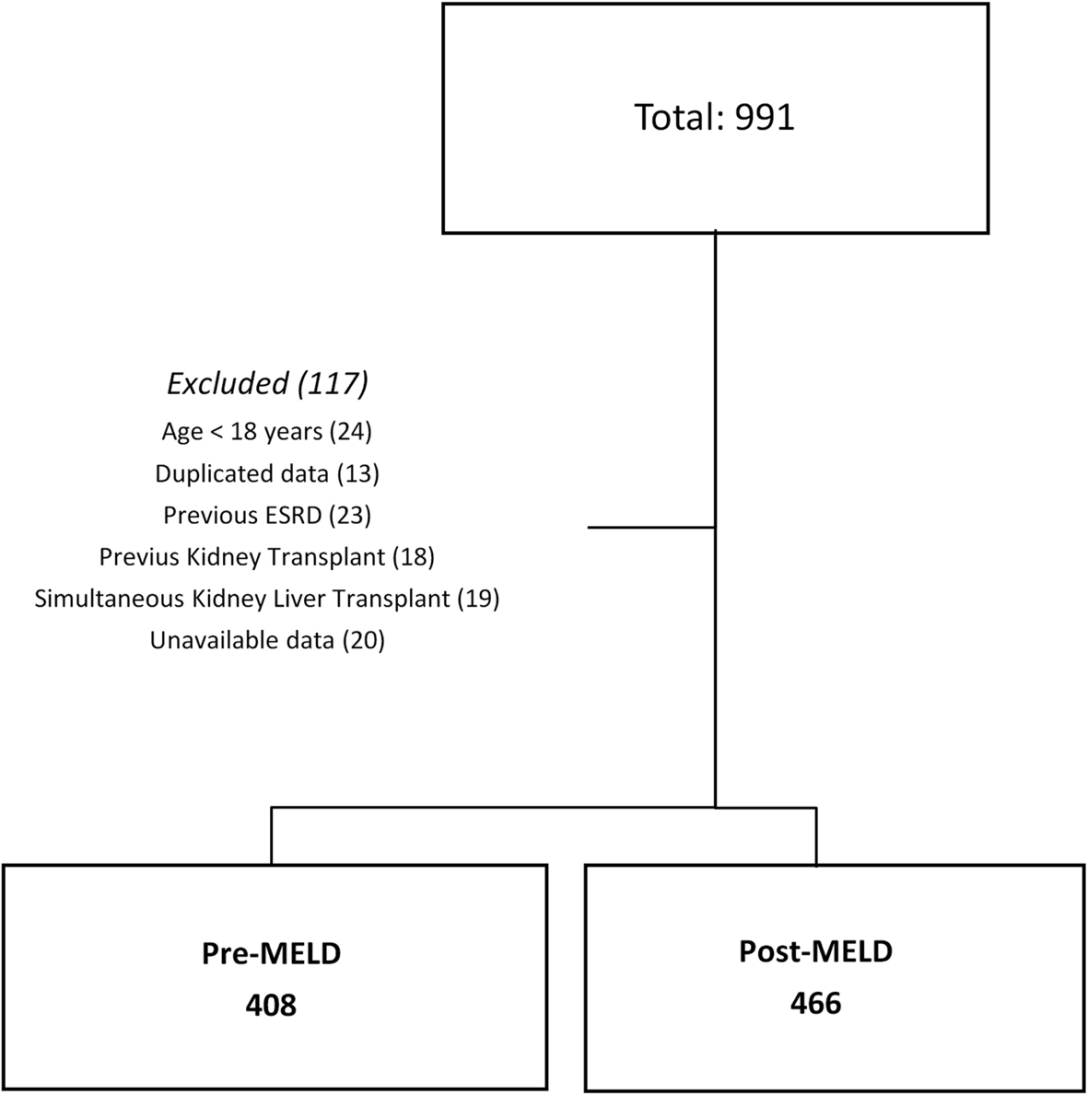
Overview of the study cohort. Flow chart shows patients screened, included, and excluded from analysis. ESDR: End-stage renal disease

Among 991 patients, 874 were included in final analysis, 408 (41.7%) patients were labelled as pre-MELD group while 466 (58.3%) as post MELD group (Fig. 1). Baseline characteristics of patients are provided in Table 1 according to MELD era. Our results show no differences between age, gender, albumin level and admission eGFR between groups. Post MELD era shows a higher prevalence of comorbidities—hypertension, diabetes, hepatocellular carcinoma, and non-hepatitis virus and/or alcoholic liver disease as etiology of liver disease (Fig. 1). Furthermore, post MELD era patients have a higher BMI and calculated MELD score.

**Table 1:** Basal patient characteristics according to MELD era

	PRE-MELD ERA	POST MELD ERA	*P* value
	*N*= 408	*N*=466	
Age, years	52.5 [45.25; 60]	54 [45; 61]	0.11
Gender, male (%)	266 (65)	328 (70)	0.18
BMI (Kg/m^2^)	25.2 [22.6; 28]	25.95 [23.1; 29.8]	0.02
Hypertension (%)	31 (8)	139 (30)	< 0.01
Diabetes (%)	79 (8)	139 (30)	0.02
APACHE 2	16 [13; 20]	17 [15; 18]	0.01
SAPS 3	39 [36; 42]	39 [28; 53]	0.83
Admission eGFR (mL/min/1.73m^2^)	91.5 [72; 110]	88 [57; 125]	0.46
Cause of liver disease			
Hepatitis C (%)	190 (47)	209 (45)	0.63
Hepatitis B (%)	40 (10)	30 (6)	0.08
Alcoholic liver cirrhosis (%)	83 (20)	92 (20)	0.87
Others (%)	75(18)	122 (26)	0.01
Hepatocellular carcinoma (%)	94 (23)	180 (39)	< 0.01
Familial amyloid polyneuropathy (%)	17 (4)	22 (5)	0.74
Pre-Transplant Albumin level (g/dL)	3.1 [2.7; 3.4]	3 [2.6; 3.4]	0.14
CPT Score			< 0.01
A or non-cirrhotic (%)	70 (17)	54 (12)	
B (%)	203 (50)	209 (45)	
C (%)	135 (33)	203 (44)	
MELD score	14 [10; 18]	18 [11; 25]	< 0.01
Preoperative Creatinine (mg/dL)	0.9 [0.7; 1.07]	0.92 [0.7; 1.32]	0.01
Preoperative Total bilirubin (mg/dL)	2.6 [1.7; 4.6]	3 [1.8; 7;5]	< 0.01
Preoperative prothrombin time, INR	1.6 [1.4; 1.96]	1.7 [1.35; 2.22]	0.14
Surgical aspects			
Deceased donor (%)	252 (62)	466 (100)	< 0.01
Piggyback technique (%)	389 (95.3)	464 (99.6)	< 0.01
Operation time (h)	7.55 [6.67; 8.5]	6 [5.17; 6.94]	< 0.01
Total ischemia time (h)	6.77 (2.70; 10.15]	9.08 [ 7.87; 10.67]	< 0.01
Vasopressor (%)	40 (9.8)	315 (67.6)	< 0.01
Number of blood packs	4 [2; 8]	3 [2; 7]	0.18
Post-transplant care			
Sepsis during hospitalization (%)	157 (39)	158 (34)	0.18
Vasopressor after transplant (%)	70 (17)	264 (30)	< 0.01
Tacrolimus based immunosuppression (%)	299 (73)	459 (98.5)	< 0.01
First week Tacrolimus peak level (ng/mL)	12.6 [9.9; 15.9]	5.7 [3.9; 7.5]	< 0.01
Acute liver rejection (%)	123 (30)	69 (15)	< 0.01
Nephrotoxic exposure (%)	285 (70)	152 (30)	< 0,01
Urinary output (ml/Kg/H)	0.25 [0.15; 0.37]	0.55 [0.35; 0.83]	< 0.01

Data are expressed as median [IQR] or percentage

*BMI* Body mass index, *APACHE 2* Acute Physiology and Chronic Health Evaluation II, *SAPS 3* Simplified Acute Physiology Score III, *eGFR* Estimated Glomerular filtration rate, *MELD* Model for End-Stage Liver Disease, *CPT* Child–Pugh-Turcote, *ICU* Intensive Care Unit

About surgical procedure and post transplantation care, Pre-MELD era shows a higher proportion in living donor transplantation, lower total ischemia times and higher total procedure time. During in-hospital post-transplant care, there were no differences in sepsis diagnosis, but higher proportion of post MELD patients were needed vasopressors drugs, during and in the first week after liver transplantation. Immunosuppression protocols differs significantly between two eras—Post MELD patients were exposed mainly to a Tacrolimus based immunosuppression (Tacrolimus, Mycophenolate, and low dose steroid) and experimented lower first week tacrolimus peak concentration. Moreover, patients on post MELD era had a lower nephrotoxic drugs exposure compared to pre-MELD era (Table 1).

As primary outcome analysis, 706 patients (81%) fulfill KDIGO AKI criteria after liver transplant procedure, 297 (34%) KDIGO stage 1, 186 (21.3%) KDIGO stage 2, and 223 (25.5%) KDIGO stage 3. Among study periods, AKI were present in 342 (84%) and 364 (78%) in pre and post MELD periods, respectively (p 0.04). Pre-MELD patients KDIGO AKI classification proportion were 171 (41.9%) stage 1, 113 (27.7%) stage 2 and 58 (14.2%) stage 3, while post MELD patients were 126 (27%) stage 1, 73 (15.7%) stage 2 and 165 (35.4%) stage 3, with a higher proportion of KDIGO 2 and 3 (severe AKI) in post MELD group (*p* < 0.01, Table 3).

AKI diagnosis following liver transplantation remained more frequent in patients with lower pre-transplant albumin levels, lower estimated glomerular filtration rate, higher MELD values and higher CTP classification. Otherwise, patients with hepatocellular carcinoma diagnosis showed lower AKI proportion. Hypertension, Diabetes, higher BMI, and other causes of liver disease (mainly non-alcoholic steatohepatitis—NASH) were more prevalent in post MELD group, but they didn`t impact in AKI diagnosis.

About surgical and post LT characteristics—Higher procedure time, wider total ischemic times, sepsis diagnosis after transplantation, acute liver rejection, vasopressor exposure and nephrotoxic drug prescription were statistically more likely in patients with AKI. Immunosuppression protocol (Tacrolimus versus Cyclosporine based) and median Tacrolimus first week peak did not differ between patients with and without AKI (Table 2).

**Table 2:** Basal patient characteristics according to AKI occurrence

	NON-AKI	AKI	*P *value
	*N* = 168	*N* = 706	
Age, years	55 [44; 63]	53 [45; 60]	0.06
Gender, male (%)	114 (67.9)	480 (68)	0.97
BMI (Kg/m^2^)	25.7 [22.6; 28.5]	25.6 [22.9; 29.1]	0.38
Hypertension (%)	37 (22)	133 (18.8)	0.35
Diabetes (%)	41 (24.4)	25.6 [22.9; 29.1]	0.66
APACHE 2	17 [14; 19]	16 [14; 19]	0.90
SAPS 3	38 [30.25; 43]	39 [32; 44,25]	< 0.01
Admission eGFR (mL/min/1.73m^2^)	95.5 [76.2; 118]	90 [63; 121]	0.01
Pathogenesis of liver disease			
Hepatitis C (%)	75 (44.6)	324 (45.9)	0.77
Hepatitis B (%)	17 (10.1)	53 (7.5)	0.26
Alcoholic liver cirrhosis (%)	27 (16.1)	148 (21)	0.15
Others (%)	35 (20.8)	162 (22.9)	0.56
Hepatocellular carcinoma (%)	75 (44.6)	199 (28.2)	< 0.01
Familial amyloid polyneuropathy (%)	11 (6.5)	28 (4)	0.15
Pre-Transplant Albumin level (g/dL)	3.1 [2.9; 3.6]	3 [2.7; 3.3]	< 0.01
CPT Score			< 0.01
A or non-cirrhotic (%)	35 (20.8)	89 (12.6)	
B (%)	83 (49.4)	329 (46.6)	
C (%)	50 (29.8)	288 (40.8)	
MELD score	13 [8; 18]	16 [11; 23]	< 0.01
Preoperative Creatinine (mg/dL)	0.8 [0.7; 1.0]	0.9 [0.7; 1.2]	0.01
Preoperative Total bilirubin (mg/dL)	2.15 [1.4; 4.45]	3 [1.8; 5.9]	< 0.01
Preoperative prothrombin time, INR	1.5 [1.23; 1.9]	1.7 [1.4; 2.15]	< 0.01
Surgical aspects			
Deceased donor (%)	150 (89.3)	568 (80.5)	< 0.01
Piggyback technique (%)	161 (95.8)	692 (98)	0.09
Operation time (h)	6 [5.1; 7.31]	7 [5.67; 8]	< 0.01
Total ischemia time (h)	8.6 [6.8; 10.6]	8.3 [6.4; 10.4]	0.19
Vasopressor (%)	64 (38.1)	291 (41.2)	0.49
Number of blood packs	3 [1; 9]	4 [2; 8]	0.31
Post-transplant care			
Sepsis during hospitalization (%)	24 (14.3)	291 (41.2)	< 0.01
Vasopressor after transplant (%)	41 (24.4)	293 (41.5)	< 0.01
Tacrolimus based immunosuppression (%)	153 (91.1)	605 (85.7)	0.08
First week Tacrolimus peak level (ng/mL)	7.7 [5.7; 11.8]	8.1 [4.9; 12.5]	0.77
Acute liver rejection (%)	18 (10.7)	174 (24.6)	< 0.01
Nephrotoxic exposure (%)	56 (33.3)	381 (54)	< 0.01
Urinary output (ml/Kg/H)	0.68 [0.43; 0.97]	0.36 [0.21; 0.56]	< 0.01

Data are expressed as median [IQR] or percentage

*BMI* Body mass index, *APACHE 2* Acute Physiology and Chronic Health Evaluation II, *SAPS 3* Simplified Acute Physiology Score III, *eGFR* Estimated glomerular filtration rate

MELD score, as continuous variable, was capable to predict AKI (c statistics – 0.60 CI 95% 0.55 – 0.65 *p *value < 0,01), severe AKI (c statistics – 0.59 CI 95% 0.55 – 0.63 *p *value < 0,01) and RRT requirement (c statistics – 0.67 CI 95% 0.63 – 0.72 *p *value < 0,01) after liver transplantation. Renal replacement therapy was more frequent in post MELD group, and even continuous as intermittent therapies were more prevalent in this group (*p* 0.03 and < 0.01, respectively) (Table 3).

**Table 3:** Primary and secondary outcomes according to MELD eras

	PRE-MELD ERA	POST MELD ERA	*P *value
	*N* = 408	*N* = 466	
Hospital length (days)	15 [11; 23]	13 [8; 22]	< 0.01
ICU length (days)	3 [2; 8]	2 [1; 4]	< 0.01
Retransplant during index hospitalization (%)	36 (8.8)	36 (7.7)	0.62
Acute liver rejection (%)	123 (30)	69 (15)	< 0.01
Acute Kidney Injury (%)	342 (84)	364 (78)	0.04
KDIGO 1	171 (41.9)	126 (27)	< 0.01
KDIGO 2	113 (27.7)	73 (15.7)	< 0.01
KDIGO 3	58 (14.2)	165 (35.4)	< 0.01
Severe AKI (KDIGO 2 + 3) (%)	171 (41.8)	238 (58.2)	< 0.01
Duration of AKI (days)	16 [10; 23]	6 [2; 23]	< 0.01
Peak creatinine during AKI episode (mg/dL)	1.6 [1.1; 2.1]	1.9 [1.32; 2.69]	< 0.01
Dialysis (%)	68 (17)	131 (28)	< 0.01
Continuous therapies (%)	39 (10)	68 (15)	0.03
Intermittent therapies (%)	50 (12)	115 (28)	< 0.01
Time of RRT dependence (days)	11.5 [3; 26.75]	17.5 [6; 43.75]	< 0.01
Hospital mortality (%)	33 (8)	36 (8)	0.90
28 days mortality (%)	25 (6)	45 (10)	0.36
90 days mortality (%)	51 (13)	45 (10)	0.19
1-year mortality (%)	81 (20)	50 (11)	< 0.01

Data are expressed as median [IQR] or percentage. *ICU* Intensive care unit, *AKI* Acute Kidney Injury, *RRT* Renal replacement therapy

Between APACHE2 and SAPS3, two well-known predictors of outcome in the intensive care unit setting, only SAPS3 were capable of discriminate mortality during hospital stay, 28 and 90 days – c statistics for in-hospital mortality was 0.58 (CI 95% 0.51 – 0.66 – *p *value 0.02). Furthermore, MELD score was only capable to predict long term mortality – MELD c statistics for 90-days mortality 0.58 (CI 95% 0.51 – 0.64 – *p *value 0.01).

Remarkably, one-year mortality rates were lower in post MELD group, even with higher MELD, SAPS3, severe AKI diagnosis as RRT needs in that group (*p* < 0.01). AKI diagnosis did not impact in mortality rates (in-hospital, 28, 90 and 365 days), but the length of stay in the ICU and hospital has increased. (Table 4). Despite that, mortality rates during hospital stay, 28 and 90 days were higher in patients that require RRT – 5% versus 19%, 5% versus 16%, 7% versus 25% and 11% versus 30% (all *p *values < 0.01), respectively.

**Table 4:** Primary and secondary outcomes according to AKI diagnosis

	NON-AKI	AKI	*P *value
	*N* = 168	*N* = 706	
Hospital length (days)	9 [7; 12]	16 [11; 25]	< 0.01
ICU length (days)	2 [1; 3]	3 [2; 6]	< 0.01
Retransplant during index hospitalization (%)	10 (6)	62 (8.8)	0.23
Acute liver rejection (%)	18 (10.7)	174 (24.6)	< 0.01
Hospital mortality (%)	13 (7.7)	56 (7.9)	0.93
28 days mortality (%)	14 (8.3)	48 (6.8)	0.50
90 days mortality (%)	15 (8.9)	81 (11.5)	0.41
1-year mortality (%)	17 (10.1)	114 (16.1)	0.05

Data are expressed as median [IQR] or percentage

*ICU* Intensive care unit, *KDIGO* Kidney disease – Improving Global Outcomes, *AKI* Acute Kidney Injury, *RRT* Renal Replacement Therapy

Table 5 shows logistic regression panels for AKI, RRT and mortality. We constructed 5 models using covariates that shows clinical relevance and/or statistical significance at univariate analysis. First model used only age and gender, second model added diabetes, hypertension, body mass index and MELD score before liver transplantation – clinically relevant covariates. Third model added estimated glomerular filtration rate and model 4 and 5 included variables presents before and after liver transplantation that show *p *value below 0.1 in univariate analysis. Our models support that post MELD era reduces AKI diagnosis, mainly sinking AKI KDIGO 1 and 2 insults. Otherwise, these period augment RRT needs, even corrected by many covariates but shows a signal for mortality decline after logistic regression modelling – model 4 and 5 for 90 days mortality.

**Table 5:** Logistic regression showing unadjusted and 5 distinctive adjusting models for AKI, RRT and 28 days mortality risk between MELD eras

*Predictor*	*HR*	*95% CI*	*P-*value
AKI risk by performing transplantation in Post MELD era			
Unadjusted	0.68	0.48, 0.97	0.03
Model 1—Adjusted for age and gender	0.69	0.49, 0.97	0.03
Model 2—Adjusted for age, gender, diabetes, hypertension, BMI and MELD	0.59	0.41, 0.86	< 0.01
Model 3—Adjusted for age, gender, diabetes, hypertension, BMI, MELD and eGFR	0.60	0.41, 0.87	< 0.01
Model 4—Adjusted for age, gender, diabetes, hypertension, BMI, MELD, eGFR, Vasopressor (During and after transplant), Transfusion, First week Tacrolimus peak level and Nephrotoxic drug exposure	0.46	0.24, 0.85	0.01
Model 5—Adjusted for age, gender, diabetes, hypertension, BMI, MELD, eGFR, Vasopressor (During and after transplant), Transfusion, First week Tacrolimus peak level, Nephrotoxic drug exposure, Acute liver rejection, Sepsis and SAPS 3	0.49	0.26, 0.92	0.02
RRT risk by performing transplantation in Post MELD era			
Unadjusted	1.95	1.40, 2.71	< 0.01
Model 1—Adjusted for age and gender	1.99	1.43, 2.78	< 0.01
Model 2—Adjusted for age, gender, diabetes, hypertension, BMI and MELD	1.61	1.12, 2.32	< 0.01
Model 3—Adjusted for age, gender, diabetes, hypertension, BMI, MELD and eGFR	1.73	1.20, 2.50	< 0.01
Model 4—Adjusted for age, gender, diabetes, hypertension, BMI, MELD, eGFR, Vasopressor (During and after transplant), Transfusion, First week Tacrolimus peak level and Nephrotoxic drug exposure	1.89	0.94, 3.80	0.07
Model 5—Adjusted for age, gender, diabetes, hypertension, BMI, MELD, eGFR, Vasopressor (During and after transplant), Transfusion, First week Tacrolimus peak level, Nephrotoxic drug exposure, Acute liver rejection, Sepsis and SAPS 3	2.11	1.01, 4.43	0.04
30 days mortality risk by performing transplantation in Post MELD era			
Unadjusted	1.32	0.78, 2.23	0.29
Model 1—Adjusted for age and gender	1.35	0.79, 2.29	0.26
Model 2—Adjusted for age, gender, diabetes, hypertension, BMI and MELD	1.37	0.78, 2.40	0.26
Model 3—Adjusted for age, gender, diabetes, hypertension, BMI, MELD and eGFR	1.31	0.79, 2.44	0.25
Model 4—Adjusted for age, gender, diabetes, hypertension, BMI, MELD, eGFR, Vasopressor (During and after transplant), Transfusion, First week Tacrolimus peak level and Nephrotoxic drug exposure	0.11	0.02, 0.52	< 0.01
Model 5—Adjusted for age, gender, diabetes, hypertension, BMI, MELD, eGFR, Vasopressor (During and after transplant), Transfusion, First week Tacrolimus peak level, Nephrotoxic drug exposure, Acute liver rejection, Sepsis and SAPS 3	0.13	0.02, 0.60	< 0.01

*BMI* Body mass index, *MELD* Model for end-stage liver disease, *eGFR* Estimated glomerular filtration rate, *SAPS 3* Simplified Acute Physiology Score III

**Fig. 2:**
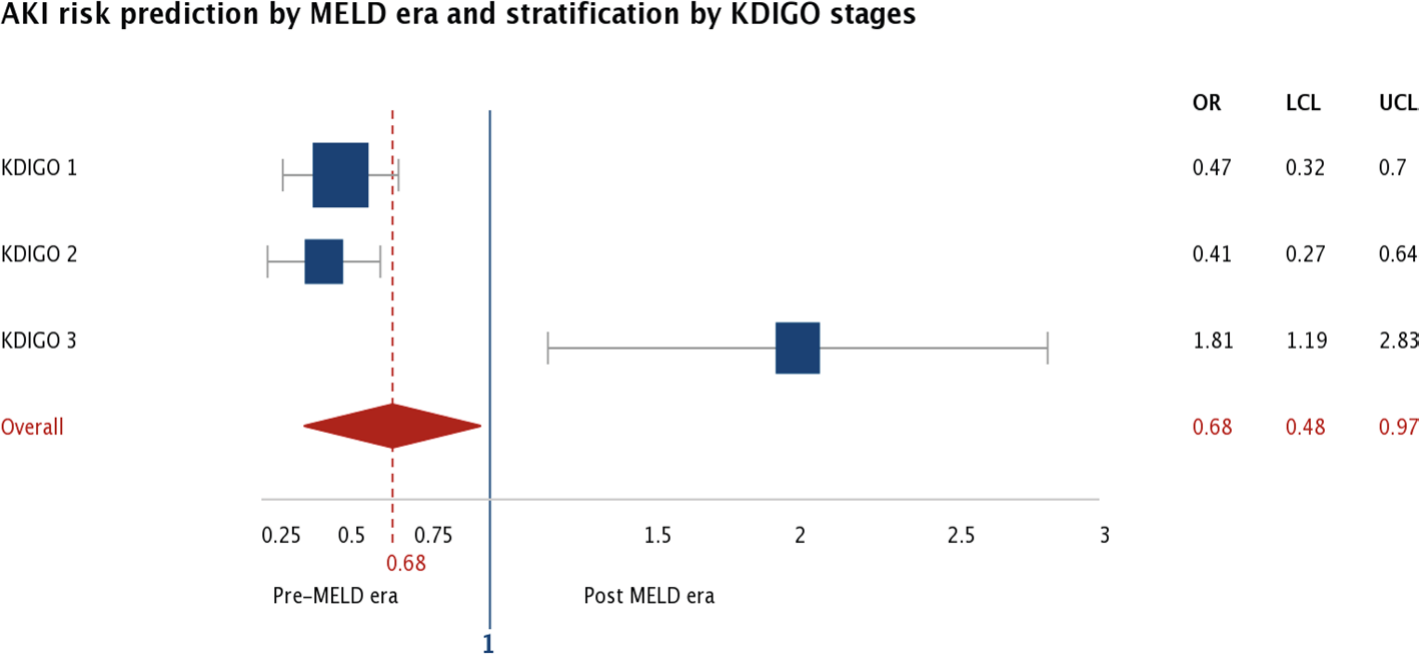
AKI risk prediction using 2 periods of time (Pre-MELD and Post MELD) and grouped by KDIGO AKI classification. AKI: Acute Kidney Injury, MELD: Model of End-Stage Liver Disease, KDIGO: Kidney disease improving global outcomes

For illustration porpoise, Fig. 2 shows each KDIGO stratum AKI risk according to MELD era group, showing that post MELD era shrinks AKI risk predominantly by KDIGO 1 and 2 reduction effect.

**Fig. 3:**
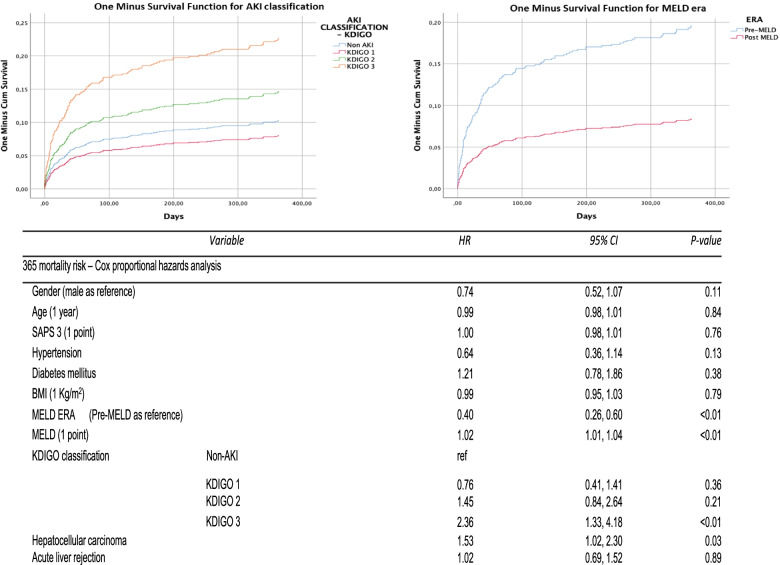
Cox proportional hazard analysis for 1-year mortality. SAPS3: Simplified Acute Physiology Score III, BMI: Body mass index, KDIGO: Kidney disease – Improving Global Outcomes, AKI: Acute Kidney Injury

Figure 3 shows a cox proportional hazard analysis for 365 days mortality risk analysis – only variables within long term prognosis impact or *p* < 0.1 in univariate analysis were included in Cox model. Liver transplantation after MELD policies implementation shows as protection factor, furthermore AKI KDIGO 3 classification impact negatively on patient survival, as MELD score at liver transplantation and hepatocellular carcinoma diagnosis.

## Discussion

As far as we know, this is the first time series that exclusively compares before and after MELD liver transplantation policy implementation influence on kidney outcomes. Our data shows that post MELD period impact in fewer AKI diagnosis, however there were more severe AKI, and these patients require more dialysis. Despite that, post MELD era shows a decline in mortality rates after 1-year period.

MELD original article from Malinchoc et al. used a Cox proportional-hazards regression model to identify variables capable to calculate a risk score to assess the short-term prognosis of patients with liver cirrhosis undergoing an elective trans = jugular intrahepatic portosystemic shunt procedure [6]. Kamath et al. validated this score as a predictor of short-term survival in patients with different liver diseases reporting that: 1) MELD score was highly predictive of 3 months mortality risk in patients with cirrhosis. 2) Inclusion of portal hypertension complications (original publication), 3) Etiology of liver disease poorly contributed to MELD predictive power [4]. After this work, MELD score was validated worldwide [5, 13, 32–35], and adopted as specific liver allocation criterion [2–6]. As MELD score stratifies patients according to their disease severity in a continuous ranking scale based exclusively on laboratorial data it appears to reinforces equality, equity and fairness in liver transplant allocation police [36], but is still under debate its use in MELD exceptions [37]. From the past 10 years, this score has been used by the UNOS, Euro transplant for prioritizing allocation of liver transplants and Brazilian liver allocation policy instead of the previous CPT score [2, 7, 8]. Recently, Godfrey et al. showed that MELD can predict 90 days mortality risk even with a decreased predictive power due to changing etiology of disease [38].

MELD allocation policy implementation impact in overall mortality has been described as neutral or positive [39–42], and liver transplant outcomes and survival benefit closely correlate with MELD score at the time of transplant [32, 43], also median time to transplant is lower in the MELD era, decreasing from 319 days in 2000 to 130 in 2020 [42]. Our data follow this trend with an increase on 1-year survival in post MELD strata.

Also, significant literature has emerged recently showing MELD potential ability to predict early morbidity – in our data, MELD was capable to predict AKI, Severe AKI and Renal Replacement therapy requirement with good performance. Romano et al. described similar results with MELD score and prediction of post LT AKI. In the same way, Park et al. described a clinical risk score system for prediction AKI after LT, in this score MELD greater than 20 points were utilized to discriminate patients [13]. Godfrey et al., in the same work previously debated, showed a higher morbidity in patients with higher MELD score [38].

Moreover, only SAPS3 were capable to discriminate in-hospital, 28 and 90 mortality rates but with a poor c-statistics score. Both APACHE2 and SAPS3 are well-known validated scores for mortality prediction [25, 26, 44–49], Serpa Neto et al*.* previously described a transition between APACHE2 and SAPS3 in general intensive care units, showing a slight better accuracy for SAPS3 in mortality prediction [50]. Also, Sakr et al. compared APACHE2 and SAPS3 as in-hospital mortality prediction in surgical ICU patients and showed that SAPS3 appeared to have the bast calibration curve on visual inspection [45]. Thereafter, APACHE2 has been previously described to overpredict mortality in different cohorts and is not useful in stratifying risk in stratum of patients within an inherently lower mortality [46–48], as in our data – APACHE2 predicted an in-hospital mortality rate higher than our actual value.

Beside, APACHE 2 and SAPS 3 usefulness for mortality prediction in transplant patients still on debate [49], de Oliveira et al. showed a lack of predictive accuracy for both SAPS3 and APACHE2 in critically ill transplant patients [51]. Another score described for mortality prediction in liver transplantation with better performance than SAPS3 and APACHE2 are APACHE4-LT [52], but unfortunately, we were unable to calculate this score for our cohort.

Additionally, we show a noticeable high incidence in AKI after liver transplantation which corroborate with previous studies that adopt KDIGO diagnostic criterion as surrogate of AKI [17]. We hypothesized that higher KDIGO sensitivity for AKI diagnosis [53], higher burden of comorbidities like hypertension and diabetes, and sicker patients, denoted by higher MELD scores [14, 22], could help to explain these proportions and were included in our logistic regression (Table 5 – Logistic regression Models 1 to 3). Also, AKI that occur in first week are usually attributed to perioperative hypotension, bleeding, nephrotoxic drug exposure, sepsis and acute liver rejection [54] (Table 5 – Logistic regression Models 4 – 5).

Surprisingly, post MELD period shows a lesser proportion in AKI diagnosis even after adjustments with 5 proposed models of logistic regression. This result could be explained by a complex interaction between dampening previous known AKI risk factors and by adopting newer strategies that could mitigate kidney damage.

Hypertension, Diabetes, higher BMI, and other causes of liver disease (mainly non-alcoholic steatohepatitis—NASH) are well-known risk factors for chronic kidney disease, and so AKI [55–57]. These risk factors were found in higher proportion in post MELD group of patients; however, we didn’t find any difference between then using AKI as independent variable.

A complex interaction between dampening previous known AKI risk factors and by adopting new strategies that could mitigate kidney damage that results in our lower prevalence of AKI in post MELD group with a higher proportion of severe AKI in these strata. A well-known AKI associated risk factor is surgical time, it`s recognized that reducing these periods of insults could mitigate kidney insult, both in general as liver transplant surgery [17, 58] – Zhou et al. showed that long operation duration (> 480 min) increased AKI risk by 6.5 times [58]. In our data, AKI patients were exposed to higher surgical time (median difference of 1 h), also post MELD group surgical time were about 2 h lower than pre-MELD group. Additionally, reduced nephrotoxic agent prescription could also support our results, summarized by protocolized sepsis treatment sparing potential nephrotoxic agents or postpone/switch radiocontrast exams by another kind of radiologic propaedeutic [59], in our data – post MELD group nephrotoxic exposure were 42% lower compared to pre-MELD group.

Additionally, adopting tacrolimus as standard post-transplant immunosuppression agent is also a previous known strategy to amend kidney injury. In addition, better tacrolimus monitoring policies could also attenuate kidney injury occurrence [60]. Unfortunately, there no differences between AKI proportion in cyclosporine and tacrolimus based immunosuppression in our data. However, in our analysis, the first week peak tacrolimus trough concentration was not found to be significantly associated with development of AKI, possibly because recipients who are suspected to occurrence AKI receive a low dose and/or late introduction of calcineurin inhibitor, as recommended by current literature [17, 54, 61]. None of these covariates individually shows impact in AKI diagnosis, but we could hypothesize that all of those together could impact in our primary endpoint. Also, combined mycophenolate protocols were found to protect against AKI, probably by permitting lower calcineurin inhibitor levels during after LT [62, 63].

Higher MELD and SAPS3 could impact in more severe AKI diagnosis and higher RRT proportions requirements, as previous described [64], also higher liver ischemic times [15, 60, 61] and more vasopressor requirement [65, 66] preclude AKI progression after liver transplantation. Sicker patients with higher vasoactive requirement and worse liver draft quality is going to experience more period and higher grade of post reperfusion syndrome [67], denoting higher risk for AKI development and progression to RRT requirement [15, 62]. In our data, AKI patients were exposed to higher proportion of vasoactive drugs during and after liver transplantation (about 6.8 and 1.7 times more), higher graft ischemic periods (an excess time about 2.3 h). Zongyi et al. showed that cold ischemic time greater than 7 h increased post-LT AKI [62] endorsing with our founds.

As previously shown, when AKI develop in post MELD patients, these diagnosis incline to worse classification categories and carries more risk for renal replacement therapy requirement, even with lower AKI diagnosis proportion in this group and lower mortality. This singularities, higher dialysis requirement with lower associated mortality, had also seen in another scenarios [68–70] and could be explained by protocolized watchfulness for kidney injury patterns reducing transient AKI episodes resulting in proportional higher persistent and severe AKI episodes [68]. Also, changing dialysis treatment patterns over the period of the study may account for reduced mortality ratios. That is, despite lack of randomized controlled data to funding this argument, perhaps earlier and higher dose and more frequent dialysis may be beneficial [71, 72]. Furthermore, Ren et al. demonstrated in retrospectively study that early continuous renal replacement therapy (CRRT) could reduce severe infection risk, length of ICU and hospital stay compared with late CRRT group [73]. Previous data from our group also shows that lower AKI exposure time (theorizing early dialysis initiation) could reduce mortality in post liver transplant patients [74]. Our logistic regression method with sequential additive models strengthens our thesis that post MELD era impact in AKI and severe AKI proportion, RRT requirement and mortality rates after liver transplantation in a tangled and wide way.

Liver transplant mortality rate is decreasing, which is encouraging, but it is important to recognize that all transformations had been made in care over the last decade resulted in this improvement over the period of study [9, 38, 75–77]. It is remarkable to note that mortality in other conditions, like acute kidney injury dialysis patients has also declined over the last decade despite the paucity of randomized trials which have shown a benefit of any specific intervention [68, 71, 72]; so, it is possible that multiple changes/bundles/improvements over period have combined to improve outcomes [78, 79].

This study has several limitations. This study has its inherent shortcomings – so only establishes association but not causality due to the retrospective study design. First, we could not determine AKI etiology in part of absence of routine urinalysis after liver transplantation. Second, liver donor characteristics to calculate donor risk index, post-reperfusion syndrome temporal data and associated variables were unavailable. Third, multiple imputation for APACHE2 and SAPS3 could prejudice they performance as mortality risk predictor. Fourth, because retrospective design itself, we couldn`t assert that liver allocation policies change was exclusively responsible for our results in AKI and one-year mortality rates reduction (about 50% in logistic and cox proportional hazards regression models, respectively Ultimately, management of liver transplantation, acute kidney injury and critically ill patient care has been improved over the period of study, likely having a significant impact in our results and conclusions). These results help to strengthen that liver transplant MELD allocation policies positively impact in non-direct correlated endpoints, like acute kidney injury.

In conclusion, MELD adoption as standard liver allocation policies reduce AKI diagnosis after liver transplantation, resulting in lower mortality rates even with higher renal replacement therapy requirement in these population.

## Data Availability

The datasets used and/or analyzed during the current study are available from the corresponding author upon reasonable request.

## Abbreviations

AKI: Acute Kidney Injury
APACHE2: Acute Physiology and Chronic Health Evaluation II
BMI: Body mass index
CMV: Cytomegalovirus
CPT: Child-Pugh-Turcotte score
CRRT: Continuous Renal Replacement Therapy
eGFR: Estimated glomerular filtration rate
HDi: Intermittent hemodialysis
ICU: Intensive Care Unit
IQR: Interquartile Range
KDIGO: Kidney Disease – Improving Global Outcomes – Acute Kidney Injury Guidelines
LT: Liver Transplantation
MDRD: Modification of Diet in Renal Disease
MELD: Model for End Stage Liver Disease
ROC: Receiver-Operating Curve
RRT: Renal Replacement Therapy
SAPS3: Simplified Acute Physiology Score III
SEVERE AKI: KDIGO 2 AND KDIGO 3 AKI
TAC: Tacrolimus
UKELD: United Kingdom – End Stage Liver Disease Score
UNOS: United Network for Organ Sharing
